# Management of the Hospitalized Transplant Patient

**DOI:** 10.1007/s11892-015-0585-6

**Published:** 2015-02-27

**Authors:** Brian Boerner, Vijay Shivaswamy, Whitney Goldner, Jennifer Larsen

**Affiliations:** 1 Division of Endocrinology and Metabolism, Department of Internal Medicine, UNMC and VA Nebraska-Western Iowa Health Care System, Omaha, NE 68105 USA; 2 987878 Nebraska Medical Center, Omaha, NE 68198-7878 USA

**Keywords:** Diabetes mellitus type 1, Diabetes mellitus type 2, Organ transplant, Kidney transplant, Heart transplant, Bone marrow transplant, Hyperglycemia, Rejection, Diabetes complication

## Abstract

Significant hyperglycemia is commonly observed immediately after solid organ and bone marrow transplant as well as with subsequent hospitalizations. Surgery and procedures are well known to cause pain and stress leading to secretion of cytokines and other hormones known to aggravate insulin action. Immunosuppression required for transplant and preexisting risk are also major factors. Glucose control improves outcomes for all hospitalized patients, including transplant patients, but is often more challenging to achieve because of frequent and sometimes unpredictable changes in immunosuppression doses, renal function, and nutrition. As a result, risk of hypoglycemia can be greater in this patient group when trying to achieve glucose control goals for hospitalized patients. Key to successful management of hyperglycemia is regular communication between the members of the care team as well as anticipating and rapidly implementing a new treatment paradigm in response to changes in immunosuppression, nutrition, renal function, or evidence of changing insulin resistance.

## Introduction

Surgeries of all kinds and post-operative pain have been long shown to increase risk of hyperglycemia by triggering cytokines and stress hormones known to aggravate insulin action [1]. Thus, it is not surprising that hyperglycemia occurs after major operations required for solid organ transplant. But, as the numbers of solid organ transplants and bone marrow transplants performed each year continue to grow, and transplant recipients live longer, more transplant patients will require management of glucose not only after their initial transplant procedure but also after subsequent hospitalizations for episodes of care that may or may not be related to their transplant. Current immunosuppression regimens contribute to hyperglycemia in organ transplant patients, as does preexisting risk.

Diabetes is common among transplant candidates, but some recipients are first recognized as having diabetes only after transplant. Diabetes that is first diagnosed after transplant, previously called New Onset Diabetes After Transplantation (NODAT), may be new onset but could also represent previously unrecognized diabetes, so a recent international consensus panel suggested changing the name to post-transplant diabetes mellitus (PTDM) [2, 3•]. Hyperglycemia is also common immediately after solid organ and bone marrow transplant, with a frequency reported at 80–90 % or more for kidney transplant recipients in the first days to week following transplant [4, 5•] but may resolve after immunosuppression doses are reduced. Thus, the diagnosis of PTDM should be reserved for hyperglycemia that persists after the recipient has been reduced to maintenance immunosuppression doses. The incidence of PTDM using these new guidelines is as yet unknown, but the increasing frequency of obesity, the significant impact of current immunosuppression regimens on insulin secretion and action, and the greater scrutiny of glucose control in all hospitalizations have led to the recognition that significant hyperglycemia is very common in the immediate post-transplant hospitalization.

With this background, whether treating preexisting diabetes, post-transplant diabetes, or new hyperglycemia that may be diagnosed as post-transplant diabetes at a later time, management of hyperglycemia in the transplant patient is common and important. This chapter will focus on the contributing factors to hyperglycemia in the hospitalized transplant patient, the current knowledge about outcomes and consequences of uncontrolled hyperglycemia after transplant, goals for glucose control, and practical strategies and considerations for glucose management in these populations, including the need to plan for care transitions to home after hospital discharge.

## Transplant-Related Factors that Contribute to Altered Glucose Metabolism in the Hospitalized Transplant Patient

### Preexisting Insulin Resistance and Glucose Intolerance, Including Post-Transplant Diabetes Mellitus

Frequency of impaired glucose tolerance and diabetes is already high in transplant populations. Diabetes is the most common cause of end-stage renal disease and need for kidney transplant in the USA [6–8]. Diabetes is also present in 14–22 % of heart transplant recipients [9–11] and up to a quarter of liver transplant recipients [12, 13•]. Obesity is also very common in transplant populations. Up to 60 % or more of kidney transplant recipients are overweight or obese at the time of transplant [14], and further weight gain after transplant is common, which can exacerbate hyperglycemia [15]. Obesity in the organ transplant recipient is directly linked to insulin resistance, as shown in kidney transplant recipients, where risk for hyperglycemia is associated with body mass index, though not necessarily in the immediate post-operative period after transplant [16, 17].

PTDM, as defined by older 2003 guidelines [2], is estimated to occur in 17 to 74 % of kidney transplant recipients, up to 30 % of liver transplant recipients, and approximately a quarter of heart transplant recipients [18–23]. Newer consensus guidelines suggest that delaying diagnosis until persistent hyperglycemia is demonstrated after the recipient has been reduced to maintenance immunosuppression doses (Table 1) [3•]. While hemoglobin A1c has been added as a diagnostic criteria for diabetes by the American Diabetes Association for diagnosis in the general population, it is associated with an unacceptable false-negative rate for diagnosis of diabetes after transplant due to the frequency of anemia and transfusion so should not be as the sole screening criteria in the first year after transplant [24]. It should be noted that even with regular screening for diabetes, transplant patients can develop interval PTDM that may remain asymptomatic and unrecognized until they are hospitalized for another cause.

**Table 1:** Diagnosis of post-transplant diabetes mellitus

Diagnosis of post-transplant diabetes mellitus is similar to the diagnosis of diabetes in non-transplant populations, where the testing should be performed outside of the hospital, with no recent history of restricted nutrition, while on maintenance immunosuppression doses. Diagnosis can be made in the following ways [3•]:
• Fasting glucose ≥126 mg/dL (7 mmol/L) on more than one occasion
• Random glucose ≥200 mg/dL (11.1 mmol/L) with symptoms
• Oral glucose tolerance test where 2 h glucose ≥200 mg/dL (11.1 mmol/L)
While hemoglobin A1C is allowed for diagnosis in non-transplant patients, it is not reliable for diagnosis of PTDM in the first year after transplant so should not be used for screening in the absence of other testing

### Immunosuppressants

One of the most significant contributing factors to hyperglycemia in the hospitalized transplant patient is the immunosuppression required by transplantation. Corticosteroids have been long recognized to contribute to hyperglycemia by causing or exacerbating insulin resistance and increasing hepatic gluconeogenesis [17, 25, 26]. High-dose corticosteroids are commonly prescribed with induction, or to treat rejection or graft-versus-host disease, as after bone marrow transplant, in addition to being part of their long-term immunosuppression for many. Other types of immunosuppressant agents also contribute to hyperglycemia including the calcineurin inhibitors (tacrolimus more than cyclosporine) and inhibitors of the mammalian target of rapamycin (mTOR), such as sirolimus. Tacrolimus can reduce insulin secretion, increase islet apoptosis, and exacerbate insulin resistance [27–30]. Calcineurin-sparing strategies have also been shown to be associated with less risk of PTDM and better graft survival in a meta-analysis of 56 randomized control trials [31•]. Sirolimus has been shown to be independently associated with PTDM, in a large cohort of kidney transplant recipients in the US Renal Data System [32]. A separate retrospective analysis of treatment regimens also demonstrated that sirolimus predisposes to PTDM after kidney transplantation [33•]. Studies in vitro show that sirolimus disrupts insulin action through multiple effects on the insulin signal transduction pathway and can reduce beta cell function and islet mass [28, 32, 34–36].

### Nutrition

Nutritional support, often required immediately after both solid organ and bone marrow transplants, can also impact glycemic control, with or without a history of diabetes [37]. Total parenteral nutrition (TPN) is required after all small bowel transplants, often needed for management of graft-versus-host disease after bone marrow transplant which affects intestinal function [38, 39], and commonly used post-transplant for other solid organ recipients when enteral nutrition is contraindicated for an extended period of time. TPN can cause hyperglycemia in all populations, and risk is greater for those with significant insulin resistance, which describes many organ transplant recipients.

### Infection

Immunosuppression increases risk for infections of all kinds after transplant [40], and severe infection, particularly sepsis, can precipitate hyperglycemia even in those patients with otherwise normal glucose tolerance [41]. Preexisting chronic infections like hepatitis C or cytomegalovirus, more common to some transplant populations, also increase risk of hyperglycemia [42].

### Improved Renal Function

Insulin requirements of diabetes patients decrease as renal disease progresses, due to reduced insulin degradation in the kidney [43]. Thus, after successful kidney transplant, insulin clearance by the kidney improves, unmasking preexisting glucose intolerance to contribute to the hyperglycemia observed in the immediate post-operative setting [44].

## Long-Term Consequences of Perioperative Glucose Control

### Patient and Graft Survival

Glucose control improves outcomes for many groups of non-transplant hospitalized patients [45–50] PTDM, whether originating before or immediately after transplant, also reduces overall graft and patient survival after kidney transplant [51, 52]. There is also increasing recognition that perioperative glycemic control alone impacts outcomes after transplant even in the absence of PTDM, possibly because of its associated risk of ischemia reperfusion and delayed graft function after kidney transplant [53•, 54•]. Graft rejection is also more frequent in kidney transplant recipients with post-operative hyperglycemia, whether or not they have diabetes [55–58]. In a retrospective review of liver and liver-kidney transplant recipients, patients with higher post-operative glucose levels (more than 200 mg/dL) had a higher rejection rate compared to those with glucose levels less than 200 mg/dL [56].

Hyperglycemia was associated with increased risk of mortality in liver transplant recipients [59]. However, to date, no studies suggest a benefit of perioperative glycemic control beyond that recommended for all hospitalized patients on mortality after kidney transplant [60•].

### Risk for Infection

In a retrospective analysis, Van den Berg et al. reported no correlation between post-operative blood glucose levels and rates of post-kidney transplant infections [61]. However, a separate retrospective analysis revealed an increased risk of rehospitalizations for infectious complications in kidney transplant recipients who developed post-operative hyperglycemia [62]. Since the rate of post-operative hyperglycemia in this population (defined as two blood glucose values >126 mg/dL) was quite low at 7.6 %, this study may not be representative of other kidney transplant populations or centers. Thomas et al. reported a significantly higher rate of infections in kidney transplant recipients who developed hyperglycemia (blood glucose >200 mg/dL) in the first 100 h post-operatively, again suggesting that post-operative hyperglycemia may be associated with increased risk for infections [57].

In liver transplant recipients, Ammori and colleagues reported a positive association between intraoperative glucose >150 mg/dL and infection rate at 30 days when compared to an intensive glucose control group (<150 mg/dL) [59]. Park and associates also reported that in patients undergoing liver transplantation, severe intraoperative hyperglycemia (>200 mg/dL) was associated with increased risk of post-operative surgical site infection when compared to those with mean blood glucose ≤180 mg/dL [63].

### Post-Transplant Diabetes Mellitus

Those with higher glucose values after transplant are also more likely to be given a diagnosis of PTDM, as suggested by a retrospective review of liver transplant recipients. Elevated fasting glucose during the first 2 weeks was also associated with a greater incidence of diabetes after transplant [64]. Conversely, early tight glycemic control after kidney transplant with insulin may actually reduce risk of later PTDM [5•].

## Glucose Goals and General Approach

Glucose goals for the hospitalized transplant patient should be the same as other hospitalized patients (ICU 140–180 mg/dL and non-ICU premeal <140 and random <180 mg/dL) [45]. The one randomized control study of kidney transplant recipients designed to assess the impact of tighter control during the immediate hospitalization (70–110 vs <180 mg/dL) did not improve any outcome, and the intensive group experienced not only more hypoglycemia but also more rejection episodes, although the latter was not statistically significant [60•]. While it might be tempting to assume that tighter control would improve outcomes after heart transplant, in particular, there are no published studies. Thus, to date, more stringent glucose control could result in worse rather than better outcomes after transplant.

Treatment of hyperglycemia immediately after transplant is generally initiated with and often requires an intravenous insulin infusion algorithm with frequent glucose monitoring. Many types of intravenous insulin protocols can successfully achieve desired glucose goals, including algorithms that are nursing-driven [65]. When the patient is ready for enteral or parenteral nutrition, the intravenous insulin infusion should be transitioned to subcutaneous insulin injections appropriate to their insulin requirements on the intravenous insulin infusion, planned size and frequency of meals, if eating, or planned parenteral or enteral tube feeding orders.

The insulin treatment regimen required at the time of transition is highly variable so does not readily lend itself to a fixed insulin per kilogram dose, in part, because of the variation in kidney function, immunosuppression regimen, corticosteroid and other immunosuppressant doses required, severity of obesity, and other factors that drive insulin resistance in this group. If the patient is being given total parenteral nutrition (TPN), 80 % of the intravenous insulin requirements over the last 24 h are usually administered as either neutral protamine Hagedorn (NPH) (1/3 delivered every 8 h) or glargine (either as a once daily dose or split with half given every 12 h). The first dose of long-acting insulin should be given at least two and preferably 4 h before the intravenous insulin is discontinued. If daily insulin requirements are decreasing rapidly, the daily requirements may be better estimated from that used in the last 8 h and multiplying by 3 to obtain the starting daily dose. After transitioning to subcutaneous insulin injections, glucose should be measured at the bedside at regular intervals, either every 4 or 6 h, with a scale provided to guide the administration of supplemental subcutaneous regular or fast-acting analog insulin. While there is no one standard, a starting point is often 1–2 units for every 40–50 mg/dL above a defined glucose threshold value, that value being 150 mg/dL in our center.

Insulin requirements can change dramatically when transitioning off TPN, so insulin requirements should either be reassessed off TPN before transitioning to subcutaneous insulin or a weight-based dose of insulin should be calculated (0.2–0.4 units/kg), of which 50 % should be used as the starting basal insulin dose, whether the patient is being started on enteral tube feeding or meals. Fast-acting insulin is then used for bolus tube feedings or for meals, usually ordered as a ratio of fast-acting insulin/grams of carbohydrates (e.g., 1–2 units/15 g as a starting dose). Again, a supplemental scale of fast-acting insulin should be provided to address blood glucose values higher than the desired range. When reliability of food ingestion is a concern, as with frequent vomiting, the dose should be given only after it is clear what the patient actually ate and kept down, not based on what is ordered.

If the patient is not on TPN and is being transitioned from clear liquids to full diet, 80 % of the intravenous insulin requirements over the last 24 h are usually given as basal insulin, most often as glargine or Levemir, with fast-acting insulin ordered for ingested food based on an insulin/ carbohydrate ratio, as described above.

An additional fixed dose of NPH or glargine (e.g., 10 units) is often added to cover intermittent corticosteroid doses given for induction or acute rejection to minimize glucose excursions from this dose alone. Whenever possible, coordinating the timing of glucocorticosteroid dose, whether planned for oral or intravenous administration, with that of the long-acting insulin will improve the ability to titrate the insulin dose to cover the possible increased requirements over that interval. When changing to subcutaneous insulin dosing, glucose testing frequency and supplemental insulin orders should also be adjusted to the timing of the subcutaneous insulin injections. Insulin doses should be adjusted daily based on the evidence of increasing or decreasing insulin resistance, planned changes in immunosuppression dosing, significant change in kidney function, or planned changes in nutrition strategy.

A multidisciplinary approach is essential to the management of transplant patients. Regular conversations between the glucose monitoring team with the nursing staff should reinforce a need for rapid communication of any planned or unplanned changes in immunosuppression; type, timing, frequency, or discontinuation of nutrition therapy; or planned surgery.

Diabetes education should be considered early, to include glucose monitoring and even insulin administration training, as intermittent use of insulin is common enough that early training may prevent a delay in discharge. When discharge is imminent, some can be considered for oral hypoglycemic agents if insulin requirements are low or to simplify therapy if there is concern that the patient or the family is having difficulty in handling glucose monitoring and/or insulin therapy after discharge. There are few available studies to assess safety or risk of most oral hypoglycemic agents in transplant patients, and there are greater potential risks for many oral agents as outlined in Table 2.

**Table 2:** Special considerations for use of oral and subcutaneous hypoglycemic agents in transplant patients

Diabetes medication class	Potential restrictions or considerations
Sulfonylureas or repaglinide	Risk of hypoglycemia if GFR is reduced, less with repaglinide than sulfonylureas; potential drug-drug interactions of sulfonylureas with cyclosporine
Metformin	Should not be used in the hospital or within 48 h of intravenous contrast administration, known heart failure, elevated liver function tests, or reduced GFR
DPP-IV inhibitors	Has not been studied with GFR <40 mL/min; linagliptin least likely to require dose adjustment for low GFR
Thiazolidinediones	May be preferentially chosen for treatment of fatty liver after liver transplant but should generally be avoided in others with elevated liver function tests, heart failure, or significant peripheral edema. May also reduce hemoglobin and bone mass
Acarbose	Avoid with low GFR; less likely to be effective for most transplant patients because of mild benefit
SGLT-2 inhibitors	No studies to assure safety in transplant patients; known to increase risk of genitourinary tract infections in women and balanitis in men so concern risk would be greater with immunosuppression. Because these agents are known to reduce GFR and can reduce blood pressure, they may have a negative impact on kidney graft
GLP-1 agonists (e.g., exenatide, liraglutide)	These agents have not been studied in transplant patients but are known to reduce intestinal motility, which may affect immunosuppressant pharmacokinetics. Should not be used with GFR <40 mL/min

There are few studies evaluating the safety or efficacy of many diabetes therapies other than insulin. While most transplant patients will be treated with insulin while in the hospital, some may be transitioned before discharge back to other agents that they were taking prior to the hospitalization. Co-morbidities should limit their use for specific patient groups. *GFR* glomerular filtration rate, *SGLT-2* sodium-glucose co-transporter-2

## Transplant-Specific Considerations

### Kidney Transplantation

Frequent and sometimes severe shifts in renal function are common in this population and can cause rapid changes in insulin requirements. This can occur in the immediate post-transplant hospitalization, as well.

### Liver Transplantation

Those being transplanted for hepatitis C, particularly if on a tacrolimus-containing regimen, are much more likely to develop significant hyperglycemia after transplant [66]. A retrospective review of patients who underwent liver transplantation concluded that post-operative infections were also lower in patients on insulin infusions managed by a glucose management service where average peri-operative glucose was 158 mg/dL, compared to those not on the glucose management service where average glucose was 189 mg/dL [67•].

### Heart Transplantation

Diabetes used to be a contraindication to heart transplant, but programs generally do not exclude diabetes outright, and there are no significant differences in outcomes between diabetic and non-diabetic recipients [68•, 69•]. However, hyperglycemia is common even in non-diabetic heart transplant recipients.

### Lung Transplantation

Many lung transplant recipients are thin, so less likely to be considered at risk for diabetes, but hyperglycemia is still common. Many patients receive lung transplant for cystic fibrosis, and many have developed cystic fibrosis-related diabetes prior to, or at risk for, hyperglycemia following transplant.

### Intestinal Transplant

It is common that patients receiving intestinal transplant require parenteral nutrition longer, but there is little data regarding the incidence of hyperglycemia or PTDM following intestinal transplant.

### Bone Marrow Transplantation

The prevalence of post-transplant hyperglycemia has been reported as high as 71 % after bone marrow transplantation (BMT) and appears to be at least partially dependent on whether or not patients received parenteral nutrition [70–72]. Hyperglycemia after BMT is associated with higher rates of complications, specifically need for red blood cell and platelet transfusion and delay in granulocyte and platelet engraftment times [72]. Hyperglycemia during the neutropenic period following myeloablative allogeneic BMT is also associated with higher rates of organ dysfunction, acute graft-versus-host disease (GVHD), and worse overall survival and non-relapse mortality [73].

Body mass index (BMI) and degree of hyperglycemia within the first 10 days after transplant appear to play a significant prognostic role for developing acute GVHD. A recent study showed that 14.2 % of normal to overweight subjects (normal BMI 21–24.9; overweight BMI 25–29.9) developed severe hyperglycemia within 10 days of allogeneic BMT (glucose >9.99 mmol/L), which significantly increased their risk of acute GVHD. More obese (BMI >30 kg/m^2^) and no lean (BMI <21 kg/m^2^) developed severe hyperglycemia, but the risk of GVHD was unrelated to severe hyperglycemia [74•].

Because skin swelling can be severe in some patients with GVHD, if subcutaneous insulin appears to be inadequately absorbed, intravenous insulin infusion may be required, as well as to achieve glucose goals in the setting of high-dose corticosteroids or with TPN. In some protocols, TPN is given as a 20-h infusion, which can be difficult to match with intravenous insulin.

## Current Strategies for Prevention of PTDM

Hyperglycemia is likely to be a risk with any large surgery and cannot be completely avoided. However, there is considerable interest in reducing severity of hyperglycemia and strategies that might reduce the longer-term risk of developing PTDM. Some of these approaches are described below.

### Choosing Immunosuppression Regimens Based on Diabetes Risk

PTDM can reduce graft and patient survival, but rejection is the number one cause of reduced graft and patient survival [75]. Since graft survival remains the most important outcome overall, the immunosuppression regimen should be selected solely based on reducing graft failure, not diabetes risk.

### Designing Immunosuppressants or Regimens with Less Risk of PTDM

Steroid-free regimens were first developed with the hope of reducing risk of PTDM but have not been associated with lower rates of PTDM because non-steroidal immunosuppressive agents also contribute to PTDM. Developing immunosuppression agents or regimens with less risk of PTDM is still a goal for many manufacturers and transplant teams.

### Preventing PTDM with Hypoglycemic Agents or Insulin

Multiple trials are in progress to determine if early introduction of hypoglycemic therapies can protect islets and prevent later PTDM, including dipeptidyl peptidase (DPP)-IV inhibitors, metformin, and insulin.

DPP-IV inhibitors and metformin are used for management of PTDM so are also attractive agents for prevention of PTDM [76•, 77•, 78•]. Thiazolidinediones (TZD) have been shown to be safe and efficacious in the management of diabetes after kidney transplantation [79, 80], but the potential side effects of TZDs have not made it an attractive prevention agent. Insulin has been studied in the prevention of PTDM, and basal insulin has been shown to decrease incidence of PTDM after kidney transplant [5•]. Metformin use can be difficult in the first year after kidney transplantation due to fluctuations in creatinine but is being studied for prevention.

### Preventing PTDM with Behavioral Interventions

Intensive lifestyle change can prevent type 2 diabetes in at risk non-transplant populations so has been suggested as a method to prevent PTDM. Active lifestyle modification including dietitian referral, exercise program, and weight loss advice benefits kidney transplant recipients with impaired glucose tolerance [81] and should be incorporated into any preventive measure.

## Summary and Conclusions

Hyperglycemia is common immediately after solid organ transplant and predicts greater risk for PTDM. Management of hyperglycemia in the immediate post-operative period is challenging because transplant recipients often have reduced or changing kidney function, experience unpredictable changes in nutrition due to nausea and vomiting and frequent need for parenteral and enteral nutrition, require treatment with types and doses of immunosuppression agents that can dramatically impact glucose intolerance, and have other events like surgery and infections or pain that can aggravate preexisting insulin resistance. Glucose goals in the hospital are the same for transplant recipients as any other hospitalized patient, but transplant recipients are at greater risk for hypoglycemia because of the frequent and potentially rapid changes in factors that impact glucose (renal function, nutrition, “stress,” and medications). It is critical that all consulting teams work together as well as with the nursing team to anticipate and rapidly respond to any changes in status that might warrant a change in intravenous insulin protocol or other pharmacologic therapy. Planning for discharge is even more important than other hospitalized patients, as ongoing changes in insulin resistance can continue to occur very rapidly. The patient needs to understand when and how to contact the team if significant changes in glucose, high or low, occur after discharge.
